# Functional and Structural Properties of Extruded Products Enriched with Mushroom-Fermented Flour

**DOI:** 10.3390/foods15193479

**Published:** 2026-09-29

**Authors:** Antonella Mazzola, Carolina Aranibar, Francisco Kuhar, Alina Greslebin, Pablo D. Ribotta

**Affiliations:** 1 Laboratorio de Bioprospección en Investigación Aplicada en Plantas y Hongos (LaBIAPH), Facultad de Ciencias Naturales y Ciencias de la Salud FCNyCS, Universidad Nacional de la Patagonia (UNPSJB), Esquel 9200, Argentina; antomazzola@outlook.com (A.M.); agreslebin@unpata.edu.ar (A.G.); 2 Instituto de Ciencia y Tecnología de los Alimentos Córdoba (ICyTAC), CONICET-UNC, Córdoba 5000, Argentina; icytac@gmail.com; 3 Instituto Multidisciplinario de Biología Vegetal (IMBIV, CONICET), Universidad Nacional de Córdoba, Córdoba 5000, Argentina; direccion.imbiv@gmail.com; 4 Facultad de Ciencias Exactas, Físicas y Naturales, Universidad Nacional de Córdoba, Córdoba 5000, Argentina

**Keywords:** solid-state fermentation, mushroom-fermented flours, snacks, extrudates, nutritional profile, phenols, riterpenes

## Abstract

Extrusion technology enables the development of nutrient-dense snacks, but most commercial extrudates rely on nutrient-poor cereal flours. This study evaluates the effect of the incorporation of mushroom-fermented flours (MFF) from *Ganoderma sessile* (GS) and *Pleurotus ostreatus* (PO), at increasing substitution levels, on the physicochemical, structural, nutritional, and functional (bioactive) properties of corn-based extrudates, relative to a corn control. Partial substitution (10–20%) significantly reduced expansion index and hardness, and reduced or maintained density, relative to the corn control, without causing structural collapse. At 100% substitution, the two species diverged sharply: GS100 retained an expansion index statistically indistinguishable from the corn control and a density numerically closer to, though still significantly different from, the corn control, while developing a markedly lower final pasting viscosity than the corn-containing formulations; PO100 produced the highest density and lowest expansion index of the entire data set, with an essentially flat pasting profile, despite both flours showing comparable shifts in protein, ash, and carbohydrate content. Incorporation of MFF markedly increased protein, ash, total phenols, triterpenes, and reducing power relative to the corn control, with the largest gains at 100% substitution; free radical-scavenging capacity increased significantly only in the 100% MFF extrudates. These results demonstrate that solid-state fermentation combined with extrusion is an effective strategy for producing nutritionally and functionally enhanced snacks.

## 1. Introduction

Extrusion technology plays a central role in the food processing industry. It contributes significantly to the production of ready-to-eat and ready-to-cook foods for humans and pets, as well as to the valorization of food by-products. This method not only provides an efficient and cost-effective approach to manufacturing a wide range of extruded products but also supports sustainability by reintegrating food processing residues into the production chain [1]. Additionally, extrusion improves the texture, digestibility, and shelf stability of food products. These features make it a key technology in large-scale food manufacturing [2].

During the extrusion process, raw materials undergo high-temperature, short-time (HTST) treatment, high absolute pressure, and intense shear forces. The characteristics of extruded snacks depend on the chemical composition, rheological behavior, and physical properties of the raw materials, as well as the operating conditions [3]. Despite their technological advantages, most extruded products on the market use nutrient-poor ingredients, such as corn flour and refined starches. These provide limited protein with a shortage of essential amino acids and scarce bioactive compounds [4]. Additionally, many ready-to-eat products are high in sugar and salt. This makes them energy-dense but nutritionally poor [4]. To address these nutritional limitations of extruded products, the incorporation of alternative ingredients rich in protein, fiber, and bioactive compounds has been explored. Adding legumes, whole grains, and edible by-products has been shown to enhance the amino acid profile, increase dietary fiber, and improve antioxidant capacity [5]. Recent studies have also noted increased interest in incorporating mushrooms into extrusion processes due to their value as functional ingredients [6,7].

Mushrooms are high in protein, dietary fiber, and bioactive compounds, which makes them valuable functional ingredients [7]. Particularly, edible and medicinal mushrooms are gaining increasing attention in food applications due to their high nutrient content, low fat, and diverse bioactive compounds, including polysaccharides, phenolics, terpenoids, and antioxidants [8,9]. Several studies have explored the incorporation of dried, sporome-derived mushroom powder into extruded formulations [7,10,11,12,13]. However, solid-state fermentation (SSF)-derived fungal flours, in which the fungus colonizes and bioconverts the substrate in situ without forming a fruiting body, have, to date, only been applied to non-extruded food matrices such as baked or steamed products [14,15,16], and a recent review of SSF applications with culinary fungi identifies thermal finishing (baking, roasting, pan-searing) as the only post-fermentation processing routes reported in the literature, with extrusion notably absent [17]. This indicates a specific gap in the use of mushroom-fermented flours, as opposed to mushroom powder, in extrusion processing. Mushroom-fermented flours (MFFs) consist of the milled dried biomass resulting from the solid-state fermentation (SSF) of plant-based substrates by filamentous fungi under aerobic conditions. This bioprocess increases protein bioavailability, enhances antioxidant capacity, and reduces antinutritional factors, all while using affordable substrates such as wheat or other cereals [18,19], as well as agricultural and food industry by-products, such as bagasse, grain husk, or bran [20]. These improvements mainly stem from the metabolic activity and biochemical composition of the fungal mycelium and depend on the fungal species used [21]. This bioconversion strategy has already been shown to enhance the nutritional and antioxidant profile of fermented flours from legumes and cereals when applied to conventional food products, including lentil flour fermented with *P. ostreatus* [15] and black bean fermented with *Ganoderma oregonense* and incorporated into steamed bread [16]; however, none of these studies subjected the fermented flour to extrusion cooking.

Powders derived from sporomes (fruiting bodies) entail high production costs and large volumes of spent substrates [22] that cannot be redirected to the food industry. In contrast, MFFs offer a potential economic advantage, avoiding the production time and cost associated with fruiting-body formation and generating no residual spent substrate; however, a direct techno-economic or life-cycle comparison between the two ingredient classes has not yet been performed and remains an identified gap for the field more broadly [17]. Extruded products use methods that bypass sporome production and enrich total biomass for inclusion in final products. Species such as *Pleurotus* spp. and *Ganoderma* spp. are particularly well-suited for food fermentation due to their enzymatic versatility, safety, and documented functional properties [23].

We have previously shown that GS- and PO-fermented whole-wheat flours retain their enhanced nutritional and bioactive profile when incorporated into a baked product (sugar-snap cookies) [14]. Extrusion, however, subjects the same fermented matrix to a fundamentally different thermomechanical environment, high shear, high-temperature short-time heating, and expansion-dependent structure formation at the die, whose effect on MFF-containing formulations has not been characterized. This study therefore extends our previous flour- and cookie-level characterization [14] to extrusion cooking specifically, addressing whether the compositional and functional gains previously demonstrated in a low-shear baked matrix persist and how they interact with the structural demands of a high-shear expanded product. Understanding these effects is essential for improving the nutritional value, physical qualities, and production efficiency of snacks. Such insights will help create snacks that balance better nutrition, high quality, and cost-effective production.

It should be noted that this study specifically targets the applied, technological question of how an already-characterized functional ingredient (MFF) behaves when incorporated into a corn-based extrudate matrix, relative to the nutrient-poor corn control that MFF incorporation is intended to improve upon [14].

This study aimed to evaluate the effect of incorporating mushroom-fermented flours (MFF) from *Ganoderma sessile* and *Pleurotus ostreatus*, at increasing substitution levels, on the physicochemical, structural, nutritional, and functional (bioactive) properties of corn-based extrudates, relative to a corn control.

## 2. Materials and Methods

### 2.1. Raw Materials

The following raw materials were used in the study: (a) Corn flour (D[4,3]: <300 µm) obtained by milling clean, sound, well-preserved corn grains free of cellulosic hulls (Grupo Alimenticio S.A., Río Cuarto, Argentina); composition: Protein 7.3%; Fat 0.20%; Ash 0.2%. This ingredient is commonly used as a raw material in the snack industry for extrusion processing. (b) Brown rice: long-grain whole-grain unpolished rice (*Oryza sativa*) with intact bran layer, purchased from the producer in the province of Entre Ríos (Villaguay, Entre Ríos, Argentina); composition: Protein 8.0%; Fat 3.0%; Ash 1.4%, db. (c) Whole wheat: hard white whole wheat (Triticum aestivum) produced in the province of Chubut (Granja de la Pradera, Las Golondrinas, Argentina); composition: Protein 9.1%; Fat 2.1%; Ash 1.6%, db.

### 2.2. Chemical Reagents

The following chemical reagents were used in the analytical procedures.: 2,2-Diphenyl-1-picrylhydrazyl (DPPH) (Sigma-Aldrich, St. Louis, MO, USA); acetic acid (Anedra, Buenos Aires, Argentina); ferric chloride (Pura Química, Córdoba, Argentina); Folin-Ciocalteu (Biopack, CABA, Argentina): gallic acid (Cicarelli, Santa Fé, Argentina); Spectrophotometric-grade hexane and methanol (Cicarelli Santa Fé, Argentina): methanol (Taurus, CABA, Argentina); perchloric acid (Dorwill, Buenos Aires, Argentina); potassium ferricyanide (Anedra, Buenos Aires, Argentina); sodium acetate (Taurus, CABA, Argentina); sodium carbonate (Anedra, Buenos Aires, Argentina); trichloroacetic acid (Cicarelli, Santa Fé, Argentina); vanillin (W310700; Sigma).

### 2.3. Strains

Two fungal strains were used: *Ganoderma sessile* (GS, strain E47, originally obtained from the University of Guelph, Guelph, ON, Canada) and *Pleurotus ostreatus* (PO, strain A02, originally obtained from CERZOS, Bahía Blanca, Argentina); both are maintained in the LaBIAPH culture collection at the National University of Patagonia (Esquel, Argentina). Stock cultures were kept and propagated on malt extract agar (MEA). To generate fresh working cultures, a 5 mm agar plug taken from the margin of an actively growing colony of each species was transferred to a 90 mm Petri dish containing MEA and incubated in darkness at 25 °C for 7 days (*G. sessile*) or 10 days (*P. ostreatus*).

### 2.4. Mushroom-Fermented Flours

Fungal spawn used as inoculum for solid-state fermentation was produced as previously described [14,21]. Briefly, brown rice was hydrated to 60% (*w*/*w*) moisture, packed in 200 g portions into gas-permeable bags, and autoclaved (121 °C/122 kPa, 45 min). Each bag was then inoculated with a single fully colonized Petri dish (~4.5 cm^2^ mycelial mat) and incubated in the dark at 25 °C for 14 days.

MFFs were obtained by solid-state fermentation of whole wheat grains, as previously described [14]. Briefly, whole wheat grains were boiled (20 min) to the target moisture content, autoclaved (121 °C/122 kPa, 90 min), aseptically inoculated with 10% (*w*/*w*) spawn suspended in sterile water, and incubated in the dark at 25 °C for 14 days. The fermented substrate was then dried at 60 °C to constant weight and milled. MFFs from ten independent trays were pooled into a single composite sample per fungal species and stored in sealed plastic bags in the dark until use.

### 2.5. Preparation of Material Supply for Extrusion

Extrusion feed materials were formulated by blending corn flour with GS-MFF or PO-MFF at 10% and 20% substitution, as detailed in Table 1. Although no prior study has combined corn flour with mushroom-fermented flour specifically, extruded products containing 5–20% (*w*/*w*) mushroom powder have been reported elsewhere [24,25]. Weighed components were mixed at ambient temperature, sealed in linear low-density polyethylene (LLDPE) bags, and held for 8 weeks at 25 °C prior to extrusion.

### 2.6. Extrusion Processing

Extrusion trials were conducted using a co-rotating Thermo Scientific Process 11 twin-screw extruder (Thermo Fisher Scientific, Karlsruhe, Germany) with a length-to-diameter (L/D) ratio of 40 and fitted with a 3 mm die. A standard three-stage screw layout incorporating two kneading blocks, a configuration routinely used for cereal-flour processing, was used throughout. Based on initial trials and operational tests, the barrel temperature profile was set from the inlet to the die section at 30, 50, 70, 100, 110, 120, 130, and 130 °C, respectively. Feed rate was held at 2.14 kg/h by a coil feeder, moisture was supplied as water at 385 mL/h (18% *w*/*w* on a flour basis) through a peristaltic pump (Watson-Marlow, Cornwall, UK) fitted with a 3.0 mm water tube, and screw speed was fixed at 600 rpm. Once the conditions were established and torque values stabilized, the extruded products were cut into approximately 3 cm pieces and dried to a constant weight in an oven at 60 °C. Two extrusion batches per formulation were obtained, each yielding approximately 500 g of extrudate product dried across several trays; the dried material from each batch’s trays was pooled and homogenized into a single composite sample per batch, which was then subsampled for physical measurement or milled for chemical analysis. A fraction of dried extrudates was milled using a hammer mill (Pulverisette^®^ 16, Fritsch, Idar-Oberstein, Germany) equipped with a 0.75 mm mesh. Dried extrudates and their milled product were packaged in sealed LLDPE bags and stored at room temperature until further analysis.

The recorded process variables were average torque, die temperature, and pressure. The specific mechanical energy input (SME) was calculated according to the following Equation (1) [26]:

(1) $$\mathrm{SME}\;(\mathrm{Wh}\;{\mathrm{kg}}^{-1})=\frac{\frac{n}{n_{\max}}\times \frac{M_{d}-M_{d,\mathrm{unload}}}{100}}{m}\times P_{\max}$$

where *n* and *n_max_* were the actual and maximum screw speeds (1000 min^−1^); *M_d_* and *M_d unload_* were the actual and idle torques (%); *m* represents the total mass flow, i.e., the combined feed rate of flour (2.14 kg·h^−1^) and water (385 mL·h^−1^, 18% *w*/*w* on a flour basis), totaling 2.53 kg·h^−1^; and *P_max_* the maximum engine power (1.5 kW).

### 2.7. Physical Properties of Extrudates

#### 2.7.1. Extrudate Dimensional Characterization

Expansion index (EI) and density were assessed once the dried extrudates were obtained. For each piece, length and diameter were recorded at three randomly chosen points with a digital caliper (Bahco, CABA, Argentina); EI was then computed as the mean extrudate diameter divided by the die diameter (3 mm). Density (g/cm^3^) was obtained from the mass-to-cylindrical-volume ratio of each piece. Both parameters are reported as the arithmetic mean across ten individually measured extrudates per treatment batch.

#### 2.7.2. Hardness of Extrudates

Hardness, the peak force needed to break a piece, was determined with a texture analyzer (Instron Universal Testing Machine, model 3342, Norwood, MA, USA) fitted with a 500 N load cell and a 15 mm cylindrical aluminum compression probe operating at 0.5 mm/s. Ten pieces (~3 cm each) per batch were positioned perpendicular to the base plate and compressed to failure; instrument control and data logging were handled through Bluehill 2^®^ software (Instron, Norwood, MA, USA).

#### 2.7.3. Water Absorption Index (WAI) and Water Solubility Index (WSI)

For this determination, 2.0 g of ground extrudate was dispersed in 25 mL of distilled water at room temperature and stirred gently on a magnetic stirrer for 30 min, followed by centrifugation (3500 rcf, 20 min) to separate the hydrated sediment from the supernatant. The sediment was oven-dried at 105 °C to constant weight and re-weighed, while the supernatant was transferred to a Petri dish and likewise evaporated to dryness at 105 °C to constant weight. Measurements were performed in duplicate for each treatment batch and averaged. WAI and WSI were then calculated using the formulas below:

(2) $$\mathrm{WAI}\;(\%)=\frac{\mathrm{weight}\;\mathrm{of}\;\mathrm{wet}\;\mathrm{sediment}}{\mathrm{weight}\;\mathrm{of}\;\mathrm{dry}\;\mathrm{sample}}\times 100$$

(3) $$\mathrm{WSI}\;(\%)=\frac{\mathrm{weight}\;\mathrm{of}\;\mathrm{dry}\;\mathrm{solid}\;\mathrm{in}\;\mathrm{supernatant}}{\mathrm{dry}\;\mathrm{weight}\;\mathrm{of}\;\mathrm{extrudate}}\times 100$$

#### 2.7.4. Pasting Profile

Pasting behavior was recorded on a Rapid Visco Analyser (RVA series 4500; Perten Instruments, Stockholm, Sweden), following the manufacturer’s pregelatinized-starch protocol (Thermocline 3.17software, Perten Instruments, Hägersten, Sweden). The flour slurry was prepared by dispersing 5.00 ± 0.01 g of the sample, 2.00 ± 0.01 g of superfine (caster) sugar, and 25.0 mL of cold (15 °C) distilled water. The pasting profile conditions were set as follows: rotation speed of 960 rpm for 10 s, followed by 160 rpm for the rest of the procedure. An initial isothermal step at 25 °C for 120 s was set, followed by a temperature increase to 95 °C within 343 s. Subsequently, the temperature was held constant at 95 °C for 3 min. The temperature was then decreased to 25 °C within 300 s and held for 300 s. The pasting profiles were determined in duplicate. During the pasting tests, the viscosity and temperature of the suspensions were recorded as a function of time. The following parameters were obtained from the curves using the Thermocline software for Windows (V 3.15, Perten Instruments, Hägersten, Sweden): cold peak (CP), final viscosity (FV), breakdown (BD), and peak time.

### 2.8. Chemical Analyses

#### 2.8.1. Preparation of Extracts

Extracts were prepared adapting the method of Puttaraju et al. [27]: 1 g of each milled-extrudate powder (dried beforehand to constant weight so that moisture content was comparable across samples) was mixed with either 5 mL distilled water or 5 mL absolute ethanol, vortexed for 30 min at room temperature (1000 rpm; XH-D vortex, Faithful Instrument, Tianjin, China), and centrifuged (2000 rcf, 15 min, room temperature; TDL80-2D).

#### 2.8.2. Chemical Characterization of Extrudates

Soluble protein and reducing-sugar contents were assayed on the water extracts described above, using 10 µL aliquots: soluble protein by the Lowry method as modified by Peterson [28], and reducing sugars by the Somogyi–Nelson reaction [29]. Moisture, ash, lipid, and crude protein (N × 6.25) were quantified by standard AACC procedures [30], each in duplicate, and total carbohydrate was obtained by difference (100 − [ash + fat + total protein]). All compositional results are reported on a dry-weight, % (*w*/*w*) basis.

##### Total Phenol Content

The phenol content of the water extracts was quantified colorimetrically with the Folin–Ciocalteu method [31]: 10 µL of water extract was combined with 200 µL of Folin–Ciocalteu reagent and 2.0 mL distilled water, followed by 800 µL of 15.9% (*w*/*v*) sodium carbonate; after a 5 min incubation at 50 °C and cooling to room temperature, absorbance was read at 765 nm (Spectrum SP-2000 spectrophotometer, Ningbo, China) against a gallic-acid calibration curve (0.0007–0.005 mg/mL), and results were expressed as mg gallic-acid equivalents per g sample. Analyses were performed in duplicate.

##### Triterpenoid Content

The triterpenoid content was determined using the colorimetric method reported by Bidegain et al. [32], with minor modifications. Briefly, 50 μL aliquots of ethanolic extract were evaporated in open tubes in an oven at 60 °C, and 0.5 mL of 70% (*v*/*v*) perchloric acid was added to each tube. The mixtures were heated in a water bath (Vicking, CABA, Argentina) at 60 °C for 10 min, then cooled to room temperature and diluted with 2.5 mL of acetic acid and 0.15 mL of 5% (*w*/*v*) vanillin (W310700; Sigma)–acetic acid solution. The absorbance was measured at 548 nm. The total triterpenoid-equivalent signal content was expressed in mg ursolic acid equivalents per g of dry matter using a calibration curve of ursolic acid (0.0006–0.005 mg/mL). Analyses were performed in duplicate.

##### Analysis of Antioxidant Activity In Vitro

(a) Potassium ferricyanide assay

Reducing power was measured by the potassium ferricyanide method of Bibi Sadeer et al. [33]. Water extract (0.5 mL) was combined with 0.5 mL phosphate buffer (0.2 M, pH 6.6) and 0.5 mL of 1% (*w*/*v*) potassium ferricyanide and incubated at 50 °C for 20 min; 0.5 mL of 10% (*w*/*v*) trichloroacetic acid, 2.5 mL deionized water, and 0.5 mL of 0.1% (*v*/*v*) ferric chloride were then added, and absorbance was read at 700 nm. Values were expressed as mg ascorbic acid equivalents per g dry weight (calibration range 0.002–0.03 mg/mL). Analyses were performed in duplicate.

(b) Scavenging ability on DPPH radical assay

Free-radical scavenging capacity was assessed by the DPPH method of Shimada et al. [34]. Ethanolic extract (10 µL) was combined with 490 µL absolute ethanol and 1 mL of ethanolic DPPH solution to give a 1.5 mL reaction volume at a final DPPH concentration of 0.1 mM; after vortexing and a 30 min dark incubation, absorbance was measured. Analyses were performed in duplicate.

### 2.9. Statistical Analysis

A one-factor experimental design was employed to evaluate the effect of the percentage of fermented flour addition on product characteristics. The factor was the percentage of fermented flour addition with 4 levels (0, 10, 20, and 100%). The response variables were: physical properties of extrudates (expansion index, hardness, water absorption, water solubility); nutritional profile (proximal composition and soluble protein and reducing sugar content); and functional attributes (polyphenol and triterpene content, antioxidant capacity, and DPPH radical scavenging activity). Each formulation was produced in two independent extrusion batches, and the response variables were evaluated per batch. Data were analyzed using analysis of variance (ANOVA) in InfoStat software (Version 13p) [35]. Treatment means were compared using Fisher’s least significant difference (LSD) test, and differences were considered significant at *p* < 0.05. The artwork was created in Excel (Microsoft 365 version).

## 3. Results and Discussion

### 3.1. Mushroom-Fermented Flour Features

The chemical composition and the functional and technological properties of mushroom-fermented wheat flours were previously described in [14]. Briefly, fermented flours showed higher crude protein, ash, soluble protein, and reducing sugar contents, and lower carbohydrate content than unfermented ones. Fat content was higher than the control in flours fermented with *G. sessile* (GS-MFF) but lower in those fermented with *P. ostreatus* (PO-MFF). Both MFFs presented higher total phenol content, reducing power, and radical-scavenging activity than the unfermented ones. The pasting profile of MFF indicated partial starch pregelatinization due to the sterilization treatment conducted before inoculation. This previous study included, in addition to the fermented flours (GS-MFF, PO-MFF), an unfermented raw whole-wheat flour control and a sterilized, non-inoculated whole-wheat control subjected to the same hydration, autoclaving, and drying treatment as the fermented substrate but without fungal inoculation. This design showed that starch pregelatinization was attributable to the sterilization step itself, occurring independently of fermentation, whereas the large increases in soluble protein (up to 438%), total phenolics, triterpenoid content, and antioxidant/radical-scavenging capacity were only observed after fungal fermentation and not in the sterilized, non-inoculated substrate. Based on this flour-level analysis, we can reliably attribute the compositional and functional (bioactive) changes reported below to fermentation rather than to whole-wheat substitution or sterilization-induced starch modification per se.

From a compositional and functional standpoint, MFF differs from unfermented whole-wheat flour, so it is hypothesized that the nutritional profile of extruded products can be improved; however, factors such as starch pre-gelatinization, increases in reduced sugars and soluble proteins, and fiber derived from fungal hyphal cell walls can affect the extrusion process and the properties of the extruded products. One additional issue to corroborate is that the phenolic and triterpenoid compounds embedded within a fiber- and protein-rich matrix (as MFF) may be resistant to thermomechanical degradation during extrusion. These composition–function relationships provide the interpretive framework used in the following sections to examine how MFF incorporation affects the processing behavior, structure, and functional quality of the resulting extrudates.

### 3.2. Extrusion Process Parameters

Both SME and die pressure are closely related system parameters. Since they are influenced by the flow behavior of the plasticized material inside the barrel, they show how process variables (temperature, moisture, screw speed), material properties, and equipment design affect the material during extrusion [36]. SME is an integral measure of the total mechanical work exerted on the material throughout the extruder barrel.

The extrusion of corn and corn–MFF samples showed that SME ranged between 139 and 179 Wh/kg, with no significant differences as a consequence of MFF incorporation at 10–20% (Table 2). A similar pattern was observed for die pressure, which ranged from 9.0 to 10.5 bar. Given that extrusion conditions were kept constant, the compositional differences introduced by 10–20% corn replacement with MFF did not significantly affect these process parameters.

In contrast, GS100 and PO100 showed significantly lower SME (*p* < 0.05) than the corn and corn–MFF samples. This can be attributed to the reduction in starch content resulting from the conversion of plant biomass into fungal biomass and CO_2_ during fermentation, since high starch content is known to produce higher-viscosity pastes that in turn increase SME. The presence of pre-gelatinized starch in GS100 and PO100 likely reinforced this effect, as pre-gelatinized starch does not require additional energy to disrupt its crystalline granular structure, reducing the shear resistance encountered by the screw throughout the barrel. This apparently contrasts with reports in which blends with a higher proportion of fiber require higher specific energy input during extrusion [37]; however, water is well established as the most potent plasticizing solvent and viscosity modifier in extrusion systems. These results suggest that, at the moisture content used in this study, the energy-lowering effect of pre-gelatinized starch counterbalanced the energy-raising effect of the higher fiber content in GS100 and PO100, resulting in an overall lower net SME.

Die pressure, on the other hand, is intrinsically linked to the dynamics of material flow and the mechanical restriction imposed by the die. Screw speed and feed rate influence how much material is pushed toward the die per unit time, while an increase in moisture acts as a lubricant, facilitating flow and reducing die pressure; nozzle design and area also directly affect flow resistance, with smaller nozzle areas restricting flow and increasing internal pressure [36,37]. Flour composition further influences die pressure by altering the rheological properties of the dough: fibers are reported to increase matrix rigidity, producing a more viscous, viscoelastic dough with greater resistance to flow, leading to material accumulation and a consequent rise in internal pressure [38]. Consistent with this, GS100 and PO100 showed higher die pressure than the corn and corn–MFF samples, which is related to their higher fiber content [14], given that moisture content was kept constant across formulations. Thus, although the pre-gelatinized starch fraction reduced the energy required for melting throughout the barrel (lowering SME), the higher fiber content of GS100/PO100 still increased flow.

### 3.3. Effect of MFFs on the Physical Characteristics of the Extrudates

Partial substitution of corn semolina with GS-MFF and PO-MFF at 10% and 20% significantly reduced the expansion index (EI) relative to the corn control (Corn: 2.04 ± 0.05 mm/mm), with GS20, PO10, and PO20 forming a statistically homogeneous group (1.76–1.82 mm/mm) and GS10 occupying an intermediate position (1.90 mm/mm) (Figure 1). At these substitution levels, both MFFs also reduced or maintained density relative to the corn control (0.56 ± 0.04 g cm^−3^), with GS10 yielding the lowest density overall (0.45 g cm^−3^).

The extrusion of 100% MFF samples exhibited strain- and concentration-dependent effects that were not proportional to the degree of corn substitution. GS100 showed an EI (1.97 ± 0.06 mm/mm) statistically indistinguishable from the corn control, interrupting the decreasing trend observed at intermediate GS substitution levels; however, its density (0.49 g cm^−3^) was significantly different from that of the corn control. In contrast, PO100 showed the lowest EI of the entire data set (1.33 ± 0.00 mm/mm, *p* < 0.05) and the highest density (1.03 ± 0.03 g cm^−3^), significantly different from every other sample, consistent with a severe structural collapse.

This change in the expansion trend within the GS series, decreasing at intermediate substitution levels but recovering at 100%, suggests that expansion at full substitution is not governed by simple dilution of native corn starch alone. Increasing protein content and the presence of pre-gelatinized starch are known to interfere with starch–starch interactions, reducing expansion ratio and increasing density in starch-based extrudates [39]. However, the chemical composition of the extrudates (Table 3) shows that protein enrichment was of comparable magnitude in both 100% MFF samples (GS100: 15.09 g/100 g; PO100: 17.22 g/100 g, vs. 7.58 g/100 g in corn), together with similarly reduced carbohydrate content (82.9 and 80.5 g/100 g, respectively). It should be noted that protein content in Table 3 was determined by total nitrogen (Kjeldahl, N × 6.25), which does not distinguish protein-derived nitrogen from non-protein nitrogen associated with fungal cell-wall structural components such as chitin [14]. Consequently, part of the apparent protein enrichment in GS100 and PO100 may reflect chitin-derived nitrogen rather than functional protein, which is consistent with, and reinforces, the interpretation below regarding species-specific cell-wall composition. The chemical composition of *Pleurotus ostreatus* and some polypore fungi [40,41] offers a plausible, though still untested, explanation for this divergence and should be confirmed with direct compositional/rheological characterization of the isolated flours.

It is noteworthy that, although the 100% corn control exhibited the highest radial expansion among directly comparable formulations, corn–MFF blends (10–20%) achieved an efficient, though comparatively lower, expansion. This reduction is likely attributable to compositional changes in the corn–MFF blends, including a lower total native starch content and a higher proportion of pre-gelatinized starch, fiber, and protein (Table 3). These modifications may promote premature coalescence and rupture of steam bubbles during extrusion, thereby limiting the extrudate’s ability to inflate and sustain a highly expanded structure. This is consistent with reports that insoluble dietary fiber causes bubbles to burst at the die exit, resulting in fewer holes, while high protein content promotes protein–starch interactions that inhibit expansion [39,42,43].

Corn–MFF extrudates showed a lower final density than the corn control (0.56 g cm^−3^) despite their slightly lower radial expansion index. This pattern would be consistent with a finer, more evenly distributed pore network trapping more air relative to the solid mass. These results emphasize that microstructural characteristics, such as cell size distribution and wall thickness, are more decisive for texture and density than radial expansion alone, consistent with reports showing that increasing levels of non-cereal flour decreased both bubble area and bubble size with no significant effect on wall thickness in pumpkin flour–corn grit extrudates [44], and that blends with higher dietary fiber content tend to produce tighter, finer cellular structures with smaller air cells [43]. The observed behavior can be linked to the functional components present in MFF, including pre-gelatinized starch, fungal polysaccharides and hydrophobins, and higher levels of protein and fiber, which collectively may stabilize the foam matrix and modulate melt rheology. However, this interpretation would require direct microstructural confirmation (SEM or X-ray micro-CT) to be established with certainty.

### 3.4. Hardness of Extrudates

The hardness of extruded products depends fundamentally on the degree of expansion and the cellular microstructure formed during processing, and its variability results from the interaction between system parameters and raw material composition.

Breaking force (hardness) decreased significantly in extrudates produced with fermented flours relative to the corn control (64.6 ± 9.2 N), which showed the highest value in the whole data set (*p* < 0.05). The corn extrudate matrix was composed almost entirely of native starch gelatinized in situ during extrusion, forming, upon cooling, a rigid, brittle gel that was intrinsically firm. In corn–MFF blends, the matrix instead comprised a mixture of native starch (from corn semolina), pre-gelatinized starch (from the sterilization step), fiber (from hyphal cell walls and wheat bran), and low-molecular sugars from fungal enzymatic activity during SSF. This composition no longer supports the formation of an equally rigid structure. Fungal components (fiber, protein) can disrupt the continuous starch network, reducing adhesion among starch molecules and creating weak points that lower overall fracture resistance, an effect analogous to that reported for rice bran inclusion in extruded corn snacks, in which fiber addition reduced hardness through limited interaction with the gelatinized starch network [45].

In addition to changes in starch and protein network continuity, the marked increase in reducing sugars observed in corn–MFF and especially in the 100% MFF samples (Table 3) may have contributed to the reduction in hardness through a plasticizing effect. Low-molecular-weight sugars are known to act as plasticizers in cereal-based extrudates, lowering the glass transition temperature (Tg) of the amorphous matrix and shifting it toward a softer, more rubbery mechanical state at a given moisture content [46]. Since reducing sugar content increased progressively with MFF substitution level, reaching its highest values in GS100 and PO100 (10.05 and 11.36 mg/g, respectively, vs. 0.95 mg/g in corn), this plasticization effect could act in parallel with, and potentially reinforce, the structural weakening attributed to reduced starch network continuity and fungal cell-wall components, contributing to the overall reduction in extrudate hardness relative to the corn control.

Notably, GS100 showed the lowest hardness in the data set (35.8 ± 7.3 N), while all corn–MFF blends and PO100 formed a statistically overlapping intermediate group (37.7–44.2 N) not significantly different from one another. This seems a relevant finding to highlight: although density is generally positively correlated with hardness in starch-based extrudates [47], PO100, despite having by far the highest density in the entire data set (1.03 g cm^−3^), did not show a correspondingly higher hardness, remaining statistically indistinguishable from the 10–20% blends. This decoupling of density and hardness suggests that the structural collapse of PO100 does not correspond to a compact, cohesive protein–starch network (of the type typically associated with increased hardness in protein/fiber-enriched extrudates), but rather to a denser yet poorly cohesive, friable matrix. It is worth noting, too, that even 10% MFF substitution was sufficient to significantly reduce hardness relative to the corn control, indicating that the loss of matrix cohesion associated with MFF incorporation occurs earlier and is more pronounced than its effect on bulk density.

On the other hand, other studies have reported the opposite trend, that fiber addition, particularly insoluble fiber, significantly increases extrudate hardness. High dietary fiber content can increase hardness through hydrogen bonding between raw material components and water, while fiber particles simultaneously act as breaking points that cause steam bubbles to burst prematurely, resulting in a denser, stiffer matrix [43,47,48]. In corn flour extrudates, increasing cellulose fiber content (1–15% *w*/*w*) increased hardness while decreasing expansion [49]. These apparently contradictory outcomes across studies likely reflect differences in fiber type, solubility, and particle size, as well as the extent to which the fiber source retains or disrupts starch–protein network continuity. In the present study, the co-occurrence of pre-gelatinized starch, hydrolyzed carbohydrates, and fungal fiber/protein components in the same flour makes it difficult to attribute the hardness reduction to any single mechanism.

Although additional studies are required to fully elucidate the underlying mechanisms, in particular, direct microstructural and rheological characterization of GS-MFF and PO-MFF, these results suggest that solid-state fermentation can selectively modify flour functionality. In particular, *G. sessile*-fermented whole-wheat flour emerges as a promising functional ingredient that significantly reduces extrudate density while maintaining an EI comparable to the corn control, making it a suitable candidate for developing novel expanded snack products; its effect on hardness, however, was the most pronounced reduction observed among all treatments and should be considered in product design.

### 3.5. Water Absorption Index (WAI) and Water Solubility Index (WSI)

The extrusion of starch-rich flours induces profound, largely irreversible changes in the physicochemical properties of the material, reflected mainly in hydration indices and rheological behavior. WAI and WSI are key functional parameters providing direct insight into the physicochemical transformations induced by extrusion and serve as predictors of final product quality [36]. WAI reflects the volume occupied by starch, proteins, and other polysaccharides after swelling in excess water, indicating the degree of starch granule cooking and gelatinization/dextrinization; a high WAI indicates that shear and heat have disrupted internal hydrogen bonds, exposing hydroxyl groups that bind water, a critical step in forming a cohesive, elastic matrix during extrusion. Conversely, WSI quantifies the quantity of soluble molecules released (simple sugars, dextrins, soluble proteins) and indicates the extent of starch degradation or dextrinization; elevated WSI values typically reflect fragmentation of amylose and amylopectin chains due to intense mechanical shearing during extrusion [50].

WAI and WSI values are shown in Figure 2. The 100% corn control had the highest WAI (612.45 ± 1.91%), statistically indistinguishable from GS10 (605.55 ± 11.53%) and PO10 (599.25 ± 1.63%). GS20 and PO20 formed a significantly lower, intermediate group (537.40–556.70%), while GS100 and PO100 showed the lowest WAI values in the data set (390.00 ± 28.85% and 404.35 ± 1.91%, respectively), with no significant difference between them. During extrusion, native starch in corn semolina undergoes complete in situ gelatinization: the granules swell irreversibly, rupture, and release amylose and amylopectin, forming a viscous, highly hydrated three-dimensional network that maximizes WAI. In corn–MFF blends, native starch is partially replaced by pre-gelatinized and hydrolyzed starch, reducing both swelling capacity and water-holding capacity and lowering WAI accordingly. Notably, the fact that GS100 and PO100 show statistically identical WAI values, despite their strongly divergent EI/density outcomes, reinforces the conclusion, discussed above, that the reduction in native starch content is comparable between the two flours and therefore cannot, by itself, explain their different expansion behavior; the explanation must lie in compositional or structural features not captured by WAI.

WSI showed a moderate value in the corn control (25.10 ± 0.42%), indicating a controlled degree of dextrinization from shear-induced partial chain breakage. Corn–MFF blends (GS10, GS20, PO10, PO20) showed a significant, progressive reduction in WSI compared to the corn control, reaching their lowest values in GS20 and PO20 (12.85–15.20%); this is consistent with the presence of pre-cooked starch reducing the degree of shear and mechanical fragmentation experienced by the remaining native starch fraction, and with reports that higher fiber content is associated with lower WSI values due to starch–fiber interactions [43]. WSI then increased again in the 100% MFF extrudates: GS100 (20.10 ± 3.25%) and, notably, PO100 (24.25 ± 0.07%), the latter being statistically indistinguishable from the corn control and significantly higher than GS100. This pattern is consistent with the markedly elevated reducing sugar content measured (GS100: 10.05 mg/g; PO100: 11.36 mg/g, vs. 0.95 mg/g in corn) (Table 3), and suggests that fungal enzyme activity during SSF hydrolyzed polysaccharides in the flours, increasing the pool of soluble solids available for release during extrusion.

### 3.6. Pasting Profile

The classical RVA pasting profile of native starch or starch-rich flours is characterized by four sequential events [51]. During the initial heating stage, viscosity remains low as intact starch granules absorb limited water. As the gelatinization temperature approaches, granules swell irreversibly, and amylose leaches into the continuous phase, producing a sharp rise to a peak viscosity at the pasting temperature. Continued heating and mechanical shear at the holding temperature (95 °C) then disrupt the swollen, fragile granules, causing viscosity to decline to a minimum; the magnitude of this decline is quantified as breakdown (BD) and reflects the shear- and heat-stability of the paste. Upon cooling, leached amylose and, to a lesser extent, amylopectin re-associate through hydrogen bonding, forming a three-dimensional gel network that raises viscosity to a final viscosity (FV); the increase from the trough to FV is defined as setback (SB) and is generally interpreted as an indicator of short-term starch retrogradation and gel-forming capacity. This classical profile assumes that the starch entering the RVA canister is still in its native, ungelatinized granular form.

In the present study, however, all flours had already undergone thermomechanical pregelatinization during extrusion (and, in the case of GS100/PO100, additional pre-extrusion sterilization). Consequently, none of the samples developed a genuine peak viscosity during the heating ramp; instead, viscosity rose immediately upon contact with cold water, since starch granules had already lost their crystalline structure and were free to hydrate and swell at room temperature [52,53]. This instantaneous viscosity was recorded as the cold peak (CP), which functionally replaces PV as the reference maximum in pregelatinized systems. BD, SB, and FV retain their conventional definitions and interpretation. Still, their magnitude in this study reflects the stability and re-association capacity of an already-fragmented, partially degraded starch network, rather than that of intact, freshly gelatinized granules [36].

RVA profiles are shown in Figure 3. The RVA profiles of corn and corn–MFF samples started with a rapid rise in viscosity as soon as the slurry was mixed, at low temperatures (below 40 °C), because gelatinized starch granules, soluble amylose/amylopectin chains, and other hygroscopic molecules hydrated and swelled immediately upon contact with water [52]. Given the temperature at maximum viscosity, it can be hypothesized that no new gelatinization occurred; there was a hydration/swelling-driven peak, where maximum volumetric occupancy by the hydrated, swollen fragments and soluble polymers occurred. Severe extrusion conditions (high shear, high temperature, low water) can depolymerize starch to such an extent that it loses its swelling power, resulting in a very low peak [36].

At the transition from the cold viscosity section to the middle section (when the temperature reaches and holds at 95 °C), viscosity decreased due to the weak, pre-hydrated network of fragments and polymers, which underwent shear-induced alignment and disintegration by the RVA paddles. Corn and corn–MFF samples showed rapid, high breakdown, likely reflecting highly dextrinized starch, consistent with the high WSI already reported for these samples. In the final section of the pasting profile, during cooling, viscosity increased due to limited re-association capacity resulting from starch degradation during extrusion. It can be hypothesized that the final structure was a weak, paste-like gel with reduced cohesivity and a poorly developed elastic network.

GS100 and PO100 extruded flours presented pasting profiles markedly different from corn-containing samples. Their curves were low and flat compared to corn and corn–MFF samples, indicating reduced swelling power. This pattern is consistent with the extensive starch depletion and structural modification expected from combined solid-state fermentation and pre-extrusion sterilization of these flours. Notably, GS100 showed a discernible rise in final viscosity during the cooling phase (FV = 1383 cP) compared to the essentially flat profile of PO100 (FV = 411 cP), indicating a measurably higher re-association capacity in GS100. One possible explanation is that GS100 retains a greater proportion of intact amylose available to form a weak network on cooling. This RVA-based observation is consistent with, though it does not directly demonstrate, the hypothesis raised earlier regarding the divergent behavior of GS100 and PO100 in expansion index, density, and hardness: it is possible that GS100 retains a greater proportion of functional, re-associating starch-derived polymers than PO100, despite the two flours showing comparable shifts in bulk protein, carbohydrate, and ash content (Table 3). Species-specific differences between *G. sessile* and *P. ostreatus*, whether in starch utilization during fermentation, fungal cell-wall polysaccharide (chitin, β-glucan) composition, cellular structure, or some combination of these factors, remain a plausible but unconfirmed explanation for their divergent physical behavior upon extrusion. Distinguishing among these non-mutually exclusive hypotheses would require direct measurement of total starch, amylose content, starch molecular weight, dietary fiber, β-glucan and chitin content, cellular structure, and the rheological properties of the isolated flours, which was beyond the scope of the present study.

The starch pasting parameters revealed that MFF incorporation drastically altered the rheological behavior of extruded flours. Corn–MFF mixtures (10–20%) showed cold peak viscosities 1.6- to 2.5-fold higher than extruded 100% corn (Corn: 2299 cP; range across blends: 3577–5853 cP). Corn melt is highly viscous during extrusion and appears to undergo substantial mechanical shear and degradation (dextrinization). In contrast, as noted above, the presence of pre-cooked starch and low molecular sugars in the MFF blends likely reduced the degree of shear and mechanical fragmentation experienced by the remaining native corn starch (relative to 100% corn), preserving part of its structural integrity; combined with the contribution of hydrated fungal biopolymers, this resulted in a higher rise in RVA viscosity as soon as the slurry was mixed.

However, the mixed networks exhibited marked thermo-mechanical instability, as evidenced by high BD values in the corn and corn–MFF samples. Although the magnitude of the initial peak partly drove the absolute breakdown values, the percentage of decomposition (%BD ranged from 85% to 91% across the corn–MFF blends, vs. 79% in corn), confirming that the structures formed in the blends were proportionally more susceptible to heat and shear, suggesting the formation of an extensive but weakly or heterogeneously interacting network.

Final viscosity (FV) values of corn and corn–MFF blends ranged between 2285 and 3362 cP, indicating that, despite differences in degradation during extrusion, the polymeric components retained meaningful re-association capacity. FV values increased with substitution level within each MFF series (GS10 < GS20; PO10 < PO20).

In contrast, GS100 and PO100 presented flat profiles with near-negligible cold peak and breakdown values, confirming that the combination of pre-extrusion sterilization and fermentation-driven starch depletion severely reduced the substrates’ capacity to develop viscosity upon hydration; nonetheless, their diverging final viscosities (see above) indicate that this depletion was not equivalent between the two fungal species.

### 3.7. Effect of MFFs on the Chemical and Nutritional Properties of Extrudates

Table 3 shows the nutritional constituents of the extrudates. Protein and ash content increased significantly as the proportion of MFFs increased in the extruded formulation compared to the corn control, with the most pronounced increases observed in the 100% MFF extrudates: crude protein (estimated from nitrogen conversion) increased up to 2.0-fold in GS100 and 2.3-fold in PO100, while ash increased up to 8.4-fold in GS100 and 11.4-fold in PO100, relative to the corn extrudate. Tepsongkroh et al. [12] similarly reported that incorporating straw mushroom (*Volvariella volvacea*) into brown rice-based extrudates increased protein and mineral content, yielding a product nutritionally comparable to soy- and egg-based proteins. Likewise, Tangsrianugul et al. [24] found that adding oyster mushroom powder (OMP) to corn-based extruded snacks increased protein content from 3.67% (control) to 6.79% (15% OMP), demonstrating that mushroom fortification enhances protein levels in extruded products.

Soluble protein content also increased significantly with increasing MFF proportion, reaching up to 14.8-fold (GS100) and 16.4-fold (PO100) the value of the corn extrudate. A similar pattern was observed for reducing sugar content, which reached up to 10.6-fold (GS100) and 12.0-fold (PO100) that of the corn extrudate.

These increases are not merely additive; they reflect a bioconversion process in which fungal metabolism assimilates the cereal substrate, concentrating essential nutrients as carbon is lost through respiration. The marked rise in soluble protein and reducing sugars indicates that extrusion, following SSF, acts on an already partially hydrolyzed matrix: fungal enzymes during SSF likely hydrolyzed complex proteins and polysaccharides into smaller, more soluble molecules, which were then further solubilized during extrusion without requiring severe degradation conditions. This pre-hydrolysis effect complements the pre-gelatinized-starch mechanism proposed earlier (see Extrusion Process Parameters) as a joint explanation for the lower SME observed in GS100 and PO100, and may also enhance the bioavailability of proteins and minerals in the final product, a potential functional advantage over extrudates made from native, unfermented flours. It is also worth mentioning that during SSF, protein modification does not only occur through enzymatic hydrolysis; the fungal mycelium assimilates part of it and produces its own polypeptides. This must also be taken into account when exploring the differences in behavior of the flours.

Lipid content increased significantly only in the 100% MFF extrudates (Table 3), remaining statistically unchanged at 10–20% substitution. This indicates that, within this substitution range, MFF inclusion does not significantly alter the fat profile or compromise extrudate stability, supporting the technological viability of partial replacement at these levels.

Carbohydrate content progressively decreased with increasing MFF level, with the largest reductions observed at 100% substitution (−10.3% in GS100 and −13.1% in PO100 relative to corn). This is consistent with fermented mushroom flour being inherently lower in carbohydrates than corn flour, as fungal metabolism consumes substrate sugars and starch during fermentation. The progressive decrease in total carbohydrate content with increasing MFF level directly correlated with the altered expansion behavior and rheological properties, as was previously described and discussed.

A critical divergence, however, occurs between GS100 and PO100 despite their broadly similar compositional shifts (comparable relative increases in protein, ash, soluble protein, and reducing sugars, and comparable loss of carbohydrate). As discussed in detail in Section 3.6, this compositional similarity, combined with the different physical outcomes already reported for expansion index, density, and hardness, indicates that bulk proximate composition alone cannot explain the opposite behavior of the two 100% MFF extrudates. The higher final viscosity observed for GS100 relative to PO100 in the RVA analysis (1383 vs. 411 cP) is consistent with GS100 retaining a greater proportion of functional, re-associating biopolymers (e.g., less-degraded amylose/amylopectin chains or specific fungal β-glucans) despite the near-total absence of native starch in both flours. A study comparing SSF by *G. lucidum* and *P. ostreatus* [40] shows that the former produced significantly higher α and β-glucans than the latter. It should be noted that this compositional evidence was obtained for *G. lucidum*—not *G. sessile*—cultivated on olive by-products rather than whole wheat, so its extrapolation to the present system is indicative at best and requires direct confirmation. Given the high hygroscopicity and viscosity of these components of the fungal extracellular matrix, their possible contribution to the observed effect cannot be ruled out. These interpretations remain some of several non-mutually exclusive hypotheses raised throughout this study, alongside the greater ash/mineral content of PO100 and the documented species-level differences in fungal cell-wall polysaccharide composition and solubility between *Ganoderma* and *Pleurotus*, none of which has yet been directly confirmed and all of which would benefit from targeted compositional and rheological characterization of the isolated MFFs in future work.

One point to be considered for future research is the microscopic composition of the fermented flours; while the behavior of starchy flours can normally be studied based on their chemical constituents, the mycelium of fungi such as *Ganoderma* spp. produce ramified, thick-walled pigmented hyphae that can withstand severe alkaline treatments and are a part of the final flour. Although their influence on the physical properties has only been documented for mycelium-based materials, we need to be aware of their potential effects on the rheology, hygroscopicity, and final consistency of the products that contain MFFs.

From a functional perspective, the total polyphenol and triterpene contents significantly increased as the proportion of MFFs increased in the extruded formulation (Table 4). Ferricyanide-reducing power (FRP) also increased as the proportion of MFFs in the extruded formulation increased. However, the free radical-scavenging capacity increased significantly only in 100% MFF samples. These observations demonstrated that both fermentation and extrusion preserved and promoted the accumulation and stability of bioactive compounds, in agreement with [54]. Additionally, the relative improvement in the antioxidant profile resulting from MFF incorporation was comparable to that achieved through the addition of black ear (*Auricularia auricula*-*judae*) and shiitake (*Lentinula edodes*) mushroom powder and lower than that achieved by the addition of white button (*Agaricus bisporus*) and porcini (*Boletus edulis*) mushroom powders [11,13].

The pronounced increase in protein, ash, total phenols and triterpenes in MFF-containing extrudates confirms that solid-state fermentation (SSF) with edible/medicinal mushrooms is an effective bio-fortification strategy. This attribution is supported by the flour-level comparison in [14], where an equivalent increase was absent in whole wheat subjected to sterilization alone and in unfermented raw whole wheat, confirming that the effect originates from fungal metabolic activity during SSF rather than from the wheat substrate or the thermal pre-treatment.

Altogether, these results emphasized the potential of mushroom-fermented flours (MFFs) as functional ingredients for developing nutritionally enhanced extruded products. Blends containing 10–20% MFF exhibited a balanced profile, characterized by moderate nutritional enrichment, significantly improved bioactive content, acceptable expansion, and optimal low-density texture. Such formulations could be ideal for creating fortified expanded snacks that combine desirable lightness and crunch with a clear functional appeal. Conversely, extrudates composed of 100% MFF represented specialized functional ingredients with distinct applications. GS100, distinguished by its exceptional triterpene and protein contents and retained expansion capacity, holds promise for the production of directly expanded bioactive snacks.

## 4. Conclusions

Incorporation of mushroom-fermented flour (MFF) from *Ganoderma sessile* or *Pleurotus ostreatus* improved the nutritional profile of corn extrudates, increasing crude protein, ash, and antioxidant compounds. At 10–20% substitution, MFF reduced expansion index and hardness and reduced or maintained density relative to the corn control, without compromising extrudate structure, supporting the potential of these blends for fortified expanded snacks. At 100% substitution, the two species diverged: PO-MFF extrudates showed the highest density and lowest expansion index of the data set, consistent with a poorly cohesive, friable matrix, whereas GS-MFF extrudates retained an expansion index statistically indistinguishable from the corn control, together with low density and the lowest hardness observed. Overall, these results indicate that solid-state fermentation combined with extrusion allows nutritional quality, bioactive content, and texture to be engineered simultaneously through fungal strain and inclusion-level selection. To our knowledge, this is the first report of a solid-state-fermentation-derived fungal flour, as distinct from fruiting-body-derived mushroom powder, being processed by extrusion; comparable fermented flours have so far been validated only in baked or steamed food matrices [14,16], which do not reproduce the high-shear, high-temperature conditions of extrusion cooking. These results also highlight the need for future sensory evaluation and bioavailability studies to define practical substitution limits for consumer applications.

## Figures and Tables

**Figure 1:**
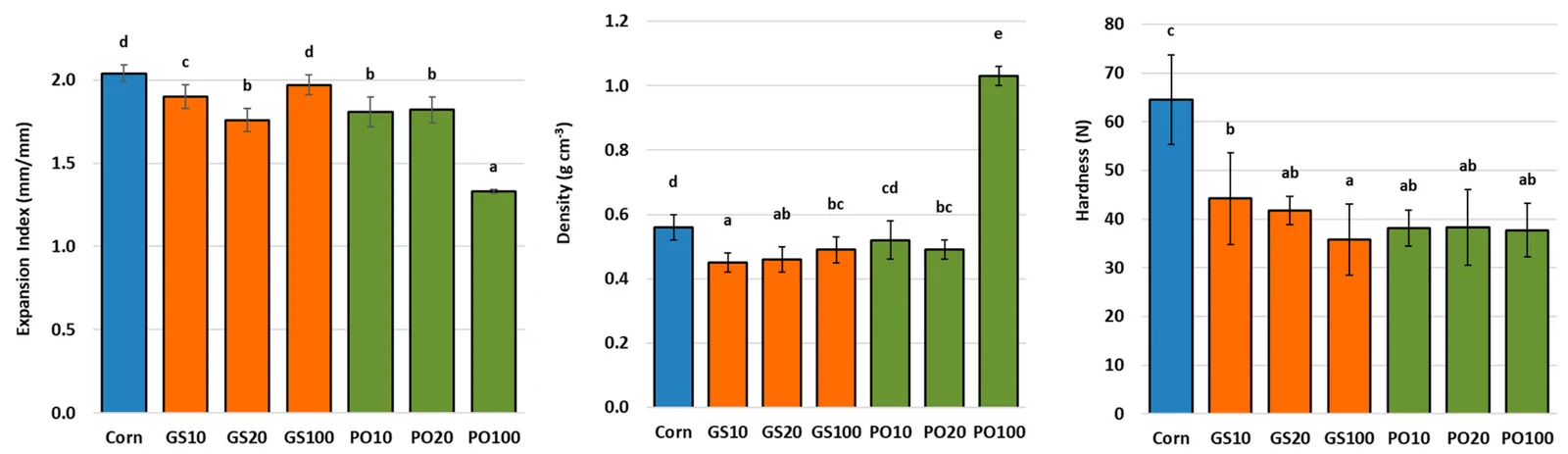
MFFs effect on expansion index, density, and hardness of extrudates. (Columns represent mean values, while error bars show standard deviation). Values with different lowercase letters differ significantly (*p* < 0.05).

**Figure 2:**
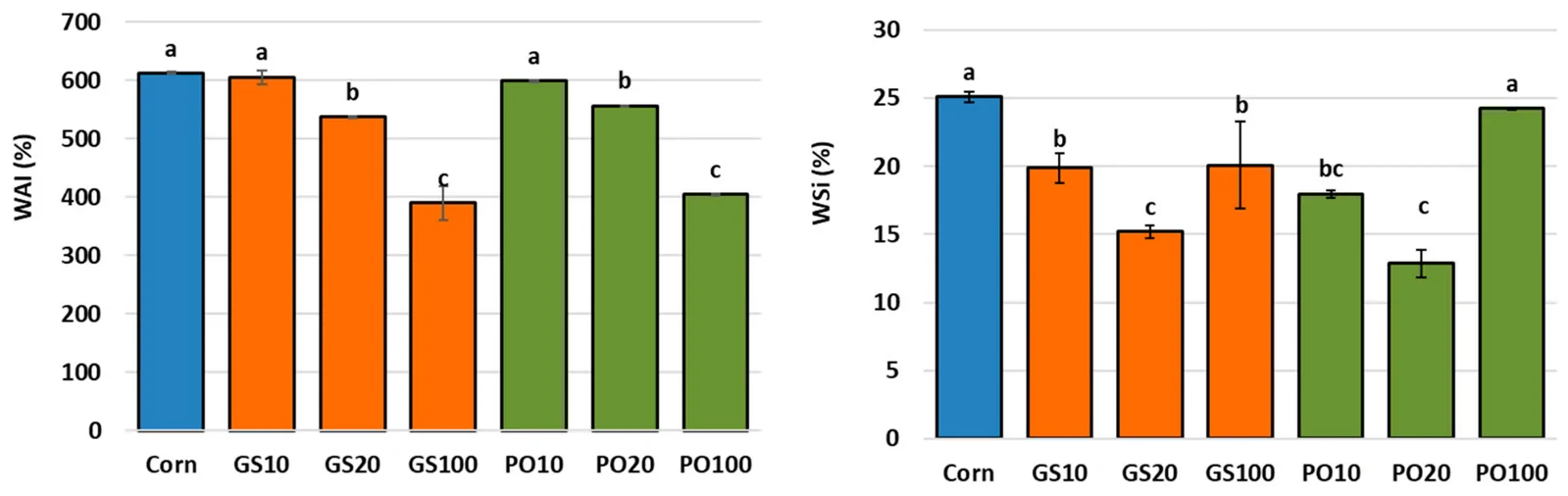
MFFs effect on the water absorption index (WAI) and water solubility index (WSI) of extrudate flours. (Data are presented as mean ± standard deviation (error bars)). Values with different lowercase letters differ significantly (*p* < 0.05).

**Figure 3:**
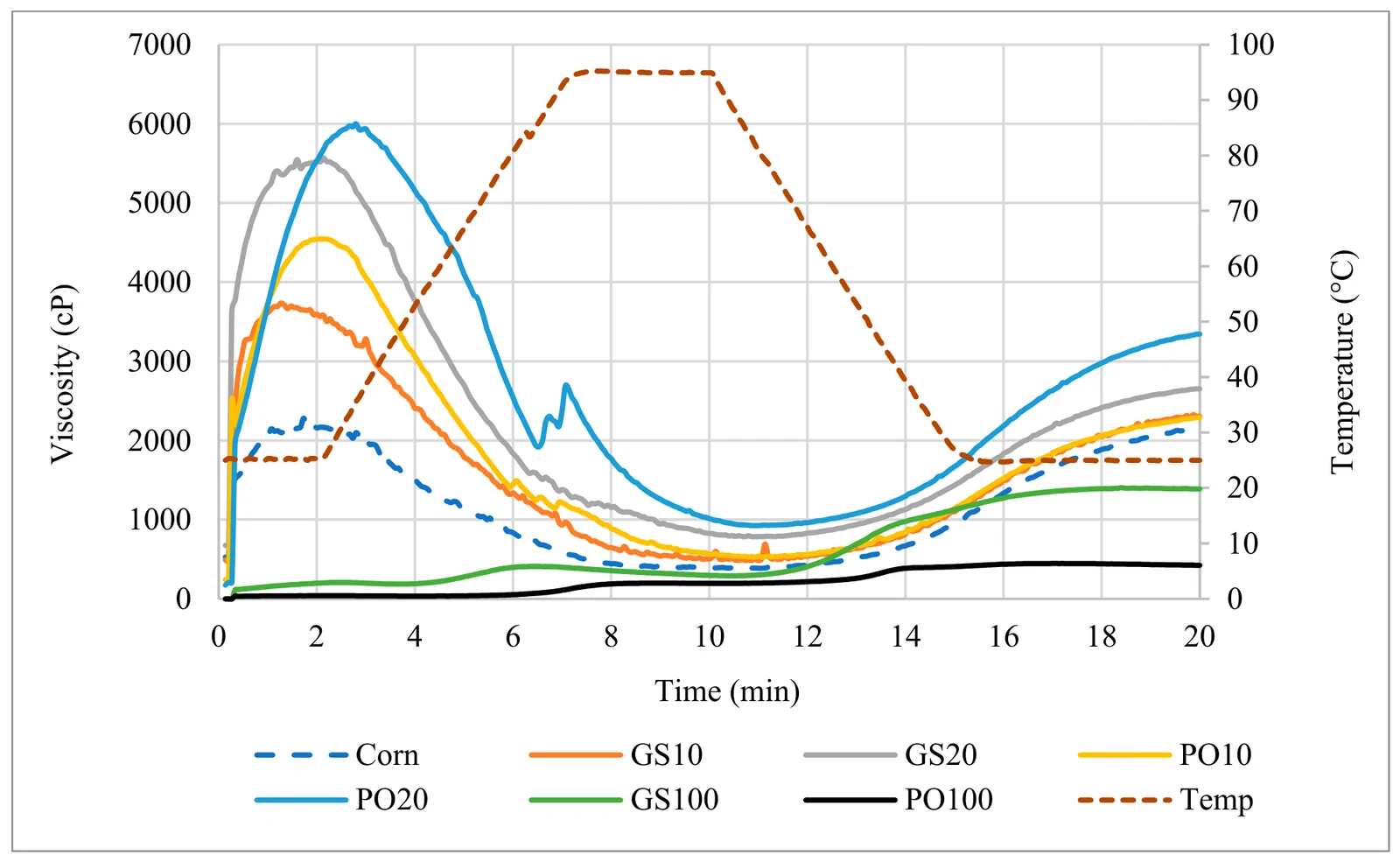
MFFs effect on the pasting profile of extrudate flours.

**Table 1:** Composition of material supply for extrusion.

Sample	Corn Flour	PO MFF	GS MFF
Corn	100%	-	-
PO10	90%	10%	-
PO20	80%	20%	-
PO100	-	100%	-
GS10	90%	-	10%
GS20	80%	-	20%
GS100	-	-	100%

100, 20, and 10 indicate the percentage of *G. sessile* (GS) or *P. ostreatus* (PO) fermented flour incorporated. MFF: mushroom-fermented flours.

**Table 2:** Extrusion process parameters of corn and corn–MFF blends.

Sample	DT (°C)	SME (Wh/kg)	DP (bar)
Corn	131 ± 1	169 ± 6	9.0 ± 0.5
GS10	130 ± 2	179 ± 7	9.0 ± 0.5
GS20	133 ± 1	177 ± 7	10.0 ± 0.5
GS100	127 ± 1	150 ± 6	12.0 ± 1.0
PO10	133 ± 1	169 ± 6	9.0 ± 0.5
PO20	132 ± 1	163 ± 6	10.5 ± 0.8
PO100	127 ± 1	139 ± 5	12.0 ± 1.5

Corn: 100% corn. 100, 20, and 10 indicate the percentage of *G. sessile* (GS) or *P. ostreatus* (PO) fermented flour incorporated. DT: die temperature, SME: specific mechanical energy, DP: die pressure.

**Table 3:** Chemical composition (expressed on a dry-matter basis) of extrudates.

Sample	Crude Protein (g/100 g)	Total Fat (g/100 g)	Ash (g/100 g)	Carbohydrate (g/100 g)	Soluble Protein (mg/g)	Reducing Sugar (mg/g)
Corn	7.58 ± 0.12 a	0.16 ± 0.01 a	0.19 ± 0.04 a	92.07	0.16 ± 0.01 a	0.95 ± 0.22 a
GS10	8.49 ± 0.16 b	0.16 ± 0.03 a	0.38 ± 0.06 b	90.97	0.76 ± 0.04 b	2.49 ± 0.62 b
GS20	9.08 ± 0.03 b	0.19 ± 0.01 a	0.53 ± 0.01 cd	90.20	1.28 ± 0.14 c	4.58 ± 0.50 c
GS100	15.09 ± 0.47 d	0.70 ± 0.10 b	1.59 ± 0.05 e	82.62	2.37 ± 0.15 e	10.05 ± 0.10 d
PO10	8.79 ± 0.19 b	0.15 ± 0.02 a	0.43 ± 0.02 bc	90.63	0.81 ± 0.01 b	2.60 ± 0.20 b
PO20	9.74 ± 0.53 c	0.13 ± 0.01 a	0.60 ± 0.02 d	89.53	1.89 ± 0.27 d	4.42 ± 0.40 c
PO100	17.22 ± 0.23 e	0.63 ± 0.03 b	2.16 ± 0.12 f	79.99	2.62 ± 0.45 e	11.36 ± 0.01 e

Corn: 100% corn. 100, 20, and 10 indicate the percentage of *G. sessile* (GS) or *P. ostreatus* (PO) fermented flour incorporated. Values in the same column followed by different lowercase letters differ significantly (*p* < 0.05). Carbohydrate content was calculated by difference and, as it is not an independently measured variable, was not subjected to a separate statistical comparison.

**Table 4:** Antioxidant profile and functional components of extrudates.

Sample	Total Phenol (mg GA/g)	Triterpenes (mg/g)	FRP (mgAA/g)	DPPH (mgAA/g)
Corn	0.23 ± 0.02 a	0.07 ± 0.00 a	0.07 ± 0.01 a	0.06 ± 0.01 a
GS10	0.41 ± 0.03 ab	0.14 ± 0.01 b	0.20 ± 0.01 b	0.08 ± 0.00 a
GS20	0.59 ± 0.08 bc	0.22 ± 0.01 c	0.25 ± 0.01 b	0.09 ± 0.01 a
GS100	1.91 ± 0.31 e	1.27 ± 0.02 f	0.98 ± 0.01 f	0.20 ± 0.01 b
PO10	0.47 ± 0.04 ab	0.18 ± 0.00 bc	0.32 ± 0.02 c	0.06 ± 0.00 a
PO20	0.79 ± 0.11 c	0.28 ± 0.02 d	0.56 ± 0.03 d	0.08 ± 0.00 a
PO100	1.36 ± 0.01 d	0.36 ± 0.04 e	0.75 ± 0.06 e	0.22 ± 0.03 b

Corn: 100% corn. 100, 20, and 10 indicate the percentage of *G. sessile* (GS) or *P. ostreatus* (PO) fermented flour incorporated. Values in the same column followed by different lowercase letters differ significantly (*p* < 0.05).

## Data Availability

All of the original data are included in the article; further inquiries can be directed to the corresponding author.

## Abbreviations

The following abbreviations are used in this manuscript:

GS	*Ganoderma sessile*
PO	*Pleurotus ostreatus*
MFF	Mushroom-fermented flour
WAI	Water absorption index
WSI	Water solubility index
SME	Specific mechanical energy input

## References

[1] Sule S., Okafor G.I., Okoyeuzu C.F., Onah G.E. (2025). Optimization of Process Variables for Extruded Snacks from Acha-Peanut Blends Separately Enriched with Carrot and Orange-Fleshed Sweet Potato Flours. Turk. J. Agric. Food Sci. Technol..

[2] Piernas C., Popkin B.M. (2011). Increased Portion Sizes from Energy-Dense Foods Affect Total Energy Intake at Eating Occasions in US Children and Adolescents: Patterns and Trends by Age Group and Sociodemographic Characteristics, 1977–2006. Am. J. Clin. Nutr..

[3] Kantrong H., Charunuch C., Limsangouan N., Pengpinit W. (2018). Influence of Process Parameters on Physical Properties and Specific Mechanical Energy of Healthy Mushroom-Rice Snacks and Optimization of Extrusion Process Parameters Using Response Surface Methodology. J. Food Sci. Technol..

[4] Brennan M.A., Derbyshire E., Tiwari B.K., Brennan C.S. (2013). Ready-to-eat Snack Products: The Role of Extrusion Technology in Developing Consumer Acceptable and Nutritious Snacks. Int. J. Food Sci. Technol..

[5] Šárka E., Sluková M., Smrčková P. (2020). New Food Compositions to Increase the Content of Phenolic Compounds in Extrudates. Czech J. Food Sci..

[6] Pérez-Montes A., Rangel-Vargas E., Lorenzo J.M., Romero L., Santos E.M. (2021). Edible Mushrooms as a Novel Trend in the Development of Healthier Meat Products. Curr. Opin. Food Sci..

[7] Ketnawa S., Rawdkuen S. (2023). Properties of Texturized Vegetable Proteins from Edible Mushrooms by Using Single-Screw Extruder. Foods.

[8] Wani B.A., Bodha R.H., Wani A.H. (2010). Nutritional and Medicinal Importance of Mushrooms. J. Med. Plants Res..

[9] Das A.K., Nanda P.K., Dandapat P., Bandyopadhyay S., Gullón P., Sivaraman G.K., McClements D.J., Gullón B., Lorenzo J.M. (2021). Edible Mushrooms as Functional Ingredients for Development of Healthier and More Sustainable Muscle Foods: A Flexitarian Approach. Molecules.

[10] Brennan M.A., Derbyshire E., Tiwari B.K., Brennan C.S. (2012). Enrichment of Extruded Snack Products with Coproducts from Chestnut Mushroom (Agrocybe Aegerita) Production: Interactions between Dietary Fiber, Physicochemical Characteristics, and Glycemic Load. J. Agric. Food Chem..

[11] Vallée M., Lu X., Narciso J.O., Li W., Qin Y., Brennan M.A., Brennan C.S. (2017). Physical, Predictive Glycaemic Response and Antioxidative Properties of Black Ear Mushroom (Auricularia Auricula) Extrudates. Plant Foods Hum. Nutr..

[12] Tepsongkroh B., Jangchud K., Jangchud A., Charunuch C., Prinyawiwatkul W. (2019). Healthy Brown Rice-based Extrudates Containing Straw Mushrooms: Effect of Feed Moisture and Mushroom Powder Contents. J. Food Process. Preserv..

[13] Lu X., Brennan M.A., Narciso J., Guan W., Zhang J., Yuan L., Serventi L., Brennan C.S. (2020). Correlations between the Phenolic and Fibre Composition of Mushrooms and the Glycaemic and Textural Characteristics of Mushroom Enriched Extruded Products. LWT.

[14] Mazzola A., Ribotta P., Kuhar F., Quiroga F., Greslebin A. (2026). Nutritional Improvement of Sugar-Snap Cookies Supplemented with *Ganoderma Sessile* and *Pleurotus Ostreatus* Solid-State Fermentation Flours. Foods.

[15] Asensio-Grau A., Calvo-Lerma J., Heredia A., Andrés A. (2020). Enhancing the Nutritional Profile and Digestibility of Lentil Flour by Solid State Fermentation with *Pleurotus ostreatus*. Food Funct..

[16] Zhang S., Wang Z., Ren X., Bai K., Wang H., Wang Y., Deng B. (2026). Enhancing the Quality of Black Bean by *Ganoderma oregonense* Solid-State Fermentation and Its Application in Steamed Bread. Foods.

[17] Gerber S., Frost S.C., Walker A.M., Kaplan D.L. (2026). Advancing Solid-State Fermentation with Culinary Fungi for Nutrient-Dense, Minimally Processed, Whole-Foods and Resilient Food Systems—A Narrative Review. Front. Nutr..

[18] Li K., Qiao K., Xiong J., Guo H., Zhang Y. (2023). Nutritional Values and Bio-Functional Properties of Fungal Proteins: Applications in Foods as a Sustainable Source. Foods.

[19] Feng X., Ng K., Ajlouni S., Zhang P., Fang Z. (2024). Effect of Solid-State Fermentation on Plant-Sourced Proteins: A Review. Food Rev. Int..

[20] Stoffel F., Santana W.D.O., Gregolon J.G.N., Kist T.B.L., Fontana R.C., Camassola M. (2019). Production of Edible Mycoprotein Using Agroindustrial Wastes: Influence on Nutritional, Chemical and Biological Properties. Innov. Food Sci. Emerg. Technol..

[21] Mazzola A., Kuhar F., Greslebin A.G. (2024). Progression Over Time of Nutritional Content and Antioxidant Activity of Grains Fermented with the Medicinal Mushrooms *Pleurotus Ostreatus* and *Ganoderma Sessile* (Agaricomycetes). Int. J. Med. Mushrooms.

[22] Finney K.N., Ryu C., Sharifi V.N., Swithenbank J. (2009). The Reuse of Spent Mushroom Compost and Coal Tailings for Energy Recovery: Comparison of Thermal Treatment Technologies. Bioresour. Technol..

[23] Stoffel F., Santana W.D.O., Fontana R.C., Camassola M. (2021). Use of *Pleurotus Albidus* Mycoprotein Flour to Produce Cookies: Evaluation of Nutritional Enrichment and Biological Activity. Innov. Food Sci. Emerg. Technol..

[24] Tangsrianugul N., Hongsanyatham S., Kapcum C., Sungayuth N., Boonsanong N., Somprasong N., Smith S.M., Amornsakchai T., Pinyo J., Wongsagonsup R. (2023). Physicochemical and Sensory Properties of Corn Grits and Pineapple Stem Starch-based Extruded Snacks Enriched with Oyster Mushroom Powder. Int. J. Food Sci. Technol..

[25] Keerthana K., Anukiruthika T., Moses J.A., Anandharamakrishnan C. (2020). Development of Fiber-Enriched 3D Printed Snacks from Alternative Foods: A Study on Button Mushroom. J. Food Eng..

[26] Schmid V., Trabert A., Keller (Schäfer) J., Bunzel M., Karbstein H.P., Emin M.A. (2021). Functionalization of Enzymatically Treated Apple Pomace from Juice Production by Extrusion Processing. Foods.

[27] Puttaraju N.G., Venkateshaiah S.U., Dharmesh S.M., Urs S.M.N., Somasundaram R. (2006). Antioxidant Activity of Indigenous Edible Mushrooms. J. Agric. Food Chem..

[28] Peterson G.L. (1977). A Simplification of the Protein Assay Method of Lowry et al. Which Is More Generally Applicable. Anal. Biochem..

[29] Somogyi M. (1952). Notes on Sugar Determination. J. Biol. Chem..

[30] AACC International (2000). Approved Methods of the American Association of Cereal Chemists.

[31] Singleton V.L., Rossi J.A. (1965). Colorimetry of Total Phenolics with Phosphomolybdic-Phosphotungstic Acid Reagents. Am. J. Enol. Vitic..

[32] Bidegain M.A., Cubitto M.A., Curvetto N.R. (2015). Optimization of the Yield of Lingzhi or Reishi Medicinal Mushroom, *Ganoderma Lucidum* (Higher Basidiomycetes), Cultivated on a Sunflower Seed Hull Substrate Produced in Argentina: Effect of Olive Oil and Copper. Int. J. Med. Mushrooms.

[33] Bibi Sadeer N., Montesano D., Albrizio S., Zengin G., Mahomoodally M.F. (2020). The Versatility of Antioxidant Assays in Food Science and Safety—Chemistry, Applications, Strengths, and Limitations. Antioxidants.

[34] Shimada K., Fujikawa K., Yahara K., Nakamura T. (1992). Antioxidative Properties of Xanthan on the Autoxidation of Soybean Oil in Cyclodextrin Emulsion. J. Agric. Food Chem..

[35] Di Rienzo J.A., Casanoves F., Balzarini M.G., Gonzalez L., Tablada M., Robledo C.W. (2019). InfoStat.

[36] Pismag R.Y., Rivera J.D., Hoyos J.L., Bravo J.E., Roa D.F. (2024). Effect of Extrusion Cooking on Physical and Thermal Properties of Instant Flours: A Review. Front. Sustain. Food Syst..

[37] Singha P., Singh S.K., Muthukumarappan K., Krishnan P. (2018). Physicochemical and Nutritional Properties of Extrudates from Food Grade Distiller’s Dried Grains, Garbanzo Flour, and Corn Grits. Food Sci. Nutr..

[38] Monti M., Gibson M., Loureiro B.A., Sá F.C., Putarov T.C., Villaverde C., Alavi S., Carciofi A.C. (2016). Influence of Dietary Fiber on Macrostructure and Processing Traits of Extruded Dog Foods. Anim. Feed. Sci. Technol..

[39] Allen K.E., Carpenter C.E., Walsh M.K. (2007). Influence of Protein Level and Starch Type on an Extrusion-expanded Whey Product. Int. J. Food Sci. Technol..

[40] Koutrotsios G., Patsou M., Mitsou E.K., Bekiaris G., Kotsou M., Tarantilis P.A., Pletsa V., Kyriacou A., Zervakis G.I. (2019). Valorization of Olive By-Products as Substrates for the Cultivation of *Ganoderma Lucidum* and *Pleurotus Ostreatus* Mushrooms with Enhanced Functional and Prebiotic Properties. Catalysts.

[41] Pérez-Bassart Z., Fabra M.J., Martínez-Abad A., López-Rubio A. (2023). Compositional Differences of β-Glucan-Rich Extracts from Three Relevant Mushrooms Obtained through a Sequential Extraction Protocol. Food Chem..

[42] Robin F., Théoduloz C., Srichuwong S. (2015). Properties of Extruded Whole Grain Cereals and Pseudocereals Flours. Int. J. Food Sci. Technol..

[43] Yi C., Qiang N., Zhu H., Xiao Q., Li Z. (2022). Extrusion Processing: A Strategy for Improving the Functional Components, Physicochemical Properties, and Health Benefits of Whole Grains. Food Res. Int..

[44] Nor N., Carr A., Hardacre A., Brennan C. (2013). The Development of Expanded Snack Product Made from Pumpkin Flour-Corn Grits: Effect of Extrusion Conditions and Formulations on Physical Characteristics and Microstructure. Foods.

[45] Renoldi N., Peighambardoust S.H., Peressini D. (2021). The Effect of Rice Bran on Physicochemical, Textural and Glycaemic Properties of Ready-to-eat Extruded Corn Snacks. Int. J. Food Sci. Technol..

[46] Masavang S., Roudaut G., Champion D. (2019). Identification of Complex Glass Transition Phenomena by DSC in Expanded Cereal-Based Food Extrudates: Impact of Plasticization by Water and Sucrose. J. Food Eng..

[47] Boakye P.G., Okyere A.Y., Bharathi R., Murai T., Annor G.A. (2023). Physicochemical and Nutritional Properties of Extruded Products from Cereals of the Triticeae Tribe–A Review. Food Chem. Adv..

[48] Toledo V.C.S., Carvalho C.W.P., Vargas-Solórzano J.W., Ascheri J.L.R., Comettant-Rabanal R. (2020). Extrusion Cooking of Gluten-free Whole Grain Flour Blends. J. Food Process Eng..

[49] Zhao Y., Zhao C., Tang X., Zhou J., Li H., Zhang H., Liu J. (2021). Physicochemical Properties and Microstructure of Corn Flour–Cellulose Fiber Extrudates. Food Sci. Nutr..

[50] Alam M.S., Kaur J., Khaira H., Gupta K. (2016). Extrusion and Extruded Products: Changes in Quality Attributes as Affected by Extrusion Process Parameters: A Review. Crit. Rev. Food Sci. Nutr..

[51] Balet S., Guelpa A., Fox G., Manley M. (2019). Rapid Visco Analyser (RVA) as a Tool for Measuring Starch-Related Physiochemical Properties in Cereals: A Review. Food Anal. Methods.

[52] Roman L., Walker M.R., Detlor N., Best J., Martinez M.M. (2022). Pregelatinized Drum-Dried Wheat Starch of Different Swelling Behavior as Clean Label Oil Replacer in Oil-in-Water Emulsions. Foods.

[53] Ma H., Liu M., Liang Y., Zheng X., Sun L., Dang W., Li J., Li L., Liu C. (2022). Research Progress on Properties of Pre-Gelatinized Starch and Its Application in Wheat Flour Products. Grain Oil Sci. Technol..

[54] Asquieri E.R., Berrios J.D.J., Asquieri E.M.D.A.R., Pan J., De Moura E Silva A.G., Batista R.D. (2020). The Effect of Extrusion Processing on the Physicochemical and Antioxidant Properties of Fermented and Non-Fermented Jabuticaba Pomace. Polímeros.

